# Negative learning behavior in small private online courses (SPOCs): findings from PLS-SEM and fsQCA

**DOI:** 10.3389/fpsyg.2025.1634957

**Published:** 2025-12-17

**Authors:** Jing Liu, Zhisen Zhang, Xuwei Zhou, Liyan Chang

**Affiliations:** 1 School of Management, Nanjing University of Posts and Telecommunications, Nanjing, Jiangsu, China; 2 School of Economics and Management, Southeast University, Nanjing, Jiangsu, China; 3 School of Computer Science, The University of Sydney, Sydney, NSW, Australia; 4 School of History and Public Administration, Yancheng Teachers University, Yancheng, Jiangsu, China

**Keywords:** SPOC, negative learning behavior, integrated model, SEM-fsQCA, MOOC

## Abstract

**Purpose:**

This study investigates the factors leading to college students’ negative learning behavior in small private online courses (SPOCs) and offers suggestions for the optimization of SPOC design and the improvement of students’ learning efficiency in online environments.

**Design/methodology/approach:**

Using partial least squares structural equation modeling (PLS-SEM) and fuzzy-set qualitative comparative analysis (fsQCA), we analyzed 351 valid questionnaires from Chinese undergraduate students to clarify the intricate causal patterns of SPOC students’ negative learning behaviors.

**Findings:**

Perceived usefulness and attitude directly impacted students’ negative learning behaviors, while perceived ease of use, task-technology fit (TTF), individual-technology fit (ITF), online-offline fit (OOF), and SPOCs’ characteristics exert indirect effects via these mediators. This study also identified five configurations that lead to poor learning behaviors in SPOCs (e.g., Low TTF + poor course quality + weak community).

**Research implications:**

Researchers and practitioners may find the results useful in understanding the causal pathways leading to students’ negative learning behaviors in SPOCs. Additionally, these finding may be used to enhance SPOC design and improve student learning efficiency.

**Originality:**

Different from previous mainstream studies, this paper takes the unique perspective of students’ negative learning behaviors to approach the issue, and adds to the body of knowledge on SPOC education. This study also employs fsQCA technology in conjunction with SEM to improve the realism of the results and address the limitations of earlier research.

## 1 Introduction

Since their emergence in 2008, Massive Open Online Courses (MOOCs) have revolutionized higher education by enabling global access to on-demand resources, active learning, and instant feedback (Guo, 2017; Baturay, 2015). However, their transformative potential has been hindered by persistent challenges such as low completion rates and limited academic engagement (Zhang J. et al., 2019; Reich and Ruiperez-Valiente, 2019). To address these limitations, Small Private Online Courses (SPOCs)—proposed by Fox (2013) as a blended learning model—have gained traction globally. SPOCs’ curated enrollment mechanisms and instructor-mediated design effectively address the completion rate challenges prevalent in MOOCs, while maintaining digital learning flexibility (Du, 2021). Currently, SPOCs have evolved into a strategic blended learning solution adopted by a substantial majority of leading universities globally (Jiang and Yin, 2024).

Although SPOCs demonstrate significant advantages in fostering teacher-student interaction and enhancing learning outcomes, they also introduce distinct challenges, particularly regarding negative learning behaviors that threaten academic integrity and educational effectiveness. These behaviors, such as “strategic skipping” of core content and collaborative cheating during online assessments, undermine the credibility of learning outcomes and erode the trustworthiness of SPOC-based education, and ultimately erode the trust essential for sustainable online learning ecosystems. Addressing these issues is critical not only to preserving SPOCs’ pedagogical value but also to upholding the integrity of blended learning ecosystems.

Current scholarship predominantly examines influencing factors in isolation or focuses on positive outcomes like intention and satisfaction. There is remains a lack of systematic research on how technology (e.g., task-technology fit, individual-technology fit), instructor (e.g., course structure, teachers presence) and psychosocial factors interact to trigger negative behaviors. Understanding how these diverse elements conjuncturally enable or necessitate negative behavior is key to developing effective interventions. This constitutes a core original contribution of our study.

This study specifically aims to address the following two questions:

*RQ1*: What is the relationship between SPOC characteristics and students’ negative learning behavior?

*RQ2*: Which combinations of factors (technological, pedagogical) are necessarily or sufficiently for triggering students’ negative learning behaviors among college students in SPOCs?

Employing a dual-method approach on survey data from 351 Chinese undergraduates, we utilize Partial least squares structural equation modeling (PLS-SEM) to identify the key influencing factors and relationships concerning RQ1. Notably, recognizing the limitations of techniques like SEM in capturing complex, non-linear, combinatorial causality (Hasan and Bao, 2022), we employ fuzzy-set qualitative comparative analysis (fsQCA) to explore configurational effects, specifically revealing how key factors such as technology (e.g., Task-technology fit) and SPOC features combine to influence. We applied fsQCA techniques alongside SEM to address this discrepancy and provide deeper insights.

In summary, this study offers four contributions. Firstly, it develops a comprehensive model of negative learning behaviors based on SPOC characteristics, expanding beyond existing frameworks focused on satisfaction or intention. Secondly, it offers a novel, “problem-centric” perspective by focusing on the drivers of undesired outcomes. Thirdly, this study acknowledges the real situation and uses the fsQCA method to explore the configuration effects among factors, making the research results more practical and realistic. Finally, the empirical findings will assist educators in developing targeted interventions to avoid negative learning behavior in SPOCs.

The paper proceeds as follows: background data and a literature review are presented in section 2, and section 3 outlines the conceptual model, hypothesis, and proposition. Section 4 DETAILS the method and results of SEM and fsQCA. Discussion, including limitations and future research directions, appears in Section 5. A final section highlights the paper’s key conclusions.

## 2 Literature review

### 2.1 Research on students’ learning in SPOCs

SPOCs are a result of the blending of online teaching with conventional instruction in the post-MOOC era. In recent years, many researchers have combined SPOCs with the flipped classroom paradigm (Van Alten et al., 2019; Wang et al., 2024b). In such classes, teachers usually interact with students to consolidate new knowledge (An et al., 2017; Alario-Hoyos et al., 2017). As a result, SPOCs have certain MOOC characteristics as well as offline, face-to-face teaching qualities. Although recent studies affirm SPOCs’ potential to enhance engagement and knowledge mastery (Esther et al., 2022), these findings predominantly reflect positive outcomes. In recent years, many academics have conducted interviews and questionnaires to determine the factors underlying students’ SPOC learning intentions (Jiang and Liang, 2023; Nejkovic and Tosic, 2018). However, the theoretical underpinnings of student behavior in SPOCs remain fragmented and insufficiently integrated. Table 1 summarizes prior studies exploring college students’ learning intention in SPOCs.

**TABLE 1:** Recent research on SPOC learning intention.

References	Methodology	Data collection	Sample/country	Framework	Analytical approach	Key findings
Kelana et al. (2017)	Quantitative	Survey	42 undergraduate students (Indonesia)	UTAUT2	PLS-SEM	Hedonic motivation has a significant effect on behavioral intention of SPOC adoption in accounting.
Zhang L. et al. (2019)	Quantitative	Survey	371 students (China)	Extended perceived fit framework (TTF+OOF+ITF)	PLS-SEM	ITF is the most significant antecedent of individual performance expectancy, followed by OOF and TTF.
Wang K. et al. (2021)	Quantitative	Questionnaire survey	202 students at TJU (China)	Extended TAM	PLS-SEM	Compatibility, self-efficacy, and vividness emerged as critical predictors of perceived usefulness.
Hewei and Youngsook (2022)	Quantitative	Online survey	1,057 Chinese art and design majors	TAM and immersion theory	PLS-SEM	User immersion experience and learning intention are positively impacted by online courses’ professionalism, interaction, interest, and ease of use.
Shi et al. (2018)	Quantitative	Questionnaire survey	161 engineering college students (China)	N/A	Variance analysis	Students who perform better academically and have participated in self-motivated MOOCs are more likely to embrace SPOC flipped classes.
Wut et al. (2022)	Quantitative	Survey	55 final-year students and 45 first-year students	Conceptual framework (Theory of planned behavior)	PLS-SEM	Desire to engage in a flipped classroom is linked to preparedness and sense of control.
Wang et al. (2022)	Quantitative	Questionnaire survey	302 students in the School of Zhengzhou Aviation Industry Management College	Conceptual framework	PLS-SEM	Students’ learning capacity has the greatest influence on the teaching effect of SPOCs, followed by learning environment and pedagogic design.
Wang T. et al. (2021)	Quantitative	Questionnaire survey	854 students from 18 universities in China	Integrated model (TTF+ECT)	PLS-SEM	The results revealed that the overall research framework largely explained continuance intention.
Filius et al. (2018)	Qualitative	Interview	41 students in a master’s epidemiology course (Netherlands)	N/A	Descriptive statistics	Peer feedback dialogues are sparked by peer feedback rating and instruction.
Lin et al. (2023)	Quantitative	Survey	A total of 197 undergraduates (China)	Conceptual framework (Attribution theories)	PLS-SEM	Correlations among attribution variables, motivational variables, and learning achievement.

### 2.2 Related models

TAM was first introduced by Fred Davis and has become a dominant model in investigating factors affecting users’ acceptance of technology (Marangunić and Granić, 2015). In recent years, TAM has been widely used in the exploration of users’ acceptance of online courses (Wang et al., 2024a). Many researchers have applied the extended technology acceptance model (TAM) to explain students’ positive learning intentions (Hewei and Youngsook, 2022; Wang K. et al., 2021). However, this reliance on TAM presents critical limitations. First, TAM privileges individual cognitive beliefs (e.g., perceived usefulness, ease of use) while neglecting contextual and configurational dynamics that shape actual learning behaviors. Second, TAM can only address the short-term beliefs and attitudes before or after the acceptance of the object (Wu and Chen, 2017).

Others have used perceived task-technology fit (TTF) (Wut et al., 2022; Zhang L. et al., 2019). TTF, which Goodhue and Thompson proposed, is described as the degree to which a technology assists a person in carrying out their tasks (Tao et al., 2022; Spies et al., 2020). We can assist in designing technical products that better meet user demands and increase user efficiency and acceptance by evaluating the degree of fit between task requirements and technical capabilities. Yet even TTF alone offers an incomplete picture. As Zhang L. et al. (2019) have shown that, compared with TTF alone, the joint influence of TTF with perceived individual-technology fit (ITF) and perceived online-to-offline fit (OOF) can be more suitable for the SPOC research context. As a result, and to make up for the shortcomings of TAM in this respect, we attempt to construct an integrated research model of SPOC negative learning behavior that incorporates TTF. Meanwhile, many qualitative findings also have a certain reference value and can be included in the external variables.

### 2.3 Fuzzy-set qualitative comparative analysis (fsQCA)

It is well known that SEM assists researchers in capturing linear relationships and structural routes more successfully when working with latent structures and complex models (Astrachan et al., 2014). However, SEM disregards interactions between elements when dealing with real-life complexities, and which may lead to oversimplification in linear decision-making analysis. However, fsQCA can identify such equifinal paths, exposing the diversity of mechanisms behind negative behaviors (Gligor and Bozkurt, 2020). The fsQCA method was first introduced by Ragin in 2000 (Kumar et al., 2022) and has been widely used by researchers to characterize specific situations (Liu X. et al., 2023). FsQCA reveals that different combinations of conditions may lead to the same results and captures the interactions that PLS-SEM often misses. As current research in the field rarely considers the effect of combined factors (Table 1), we conduct secondary analysis with the help of fsQCA, identifying the joint factors of negative learning behavior in SPOC among Chinese college students.

## 3 Theoretical framework and hypothesis development

**FIGURE 1:**
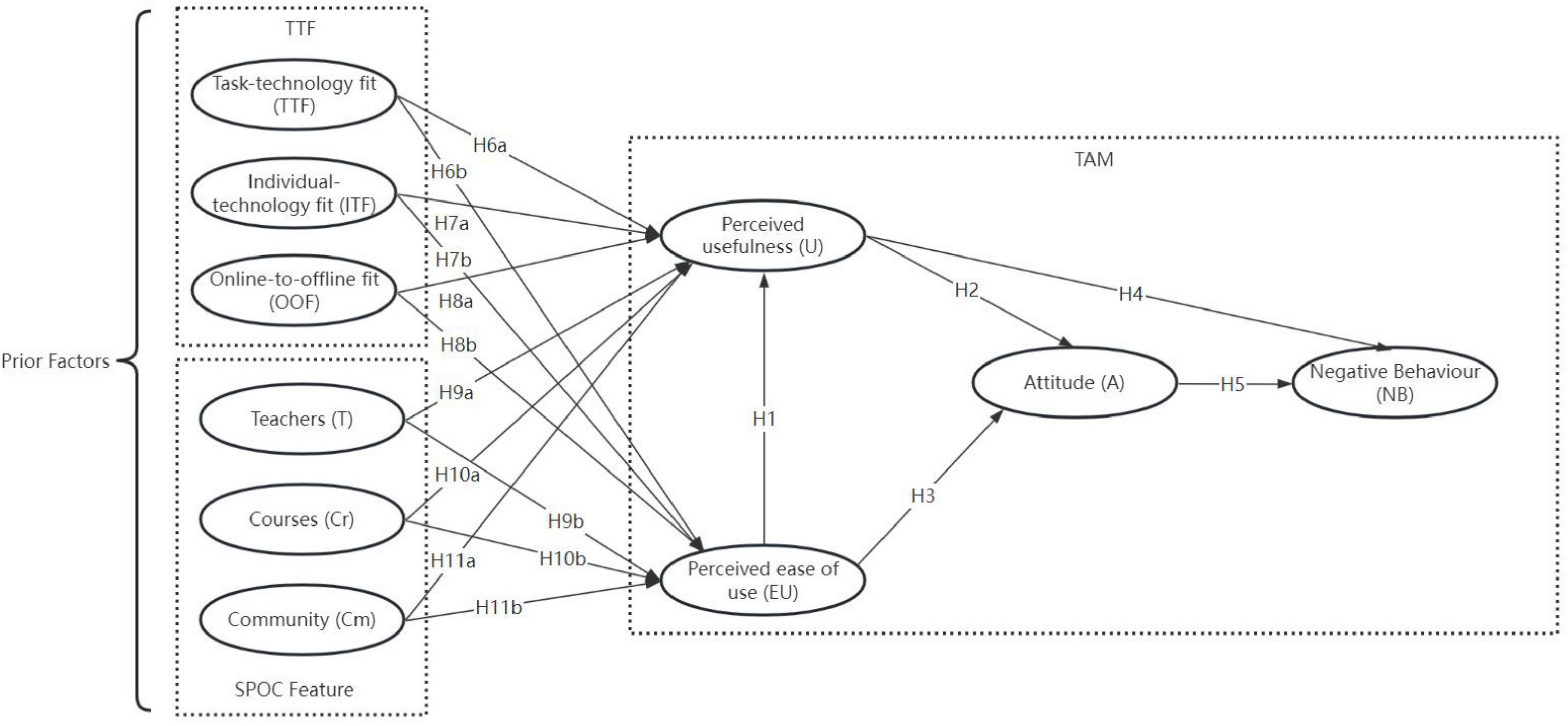
Theoretical framework.

This study developed an integrated research model based on TAM, TTF, and SPOC features. The model includes 10 variables: perceived usefulness (U), perceived ease of use (EU), attitude (A), negative behavior (NB), online-to-offline fit (OOF), individual-technology fit (ITF), task-technology fit (TTF), teachers (T), courses (Cr), and community (Cm), which are shown in Figure 1.

### 3.1 Perceived usefulness (U), perceived ease of use (EU), attitude (A), and negative behavior (NB)

U, EU and NB are basic components in TAM theory (King and He, 2006), and in this study we inherit this theory’s basic hypothesis. Specifically, U refers to how much students believe that learning SPOCs will enhance their academic performance, EU refers to the operational difficulty for students to attend SPOC courses, and NB refers to students’ negative learning behavior in SPOCs. In addition, we use a mediating variable A to evaluate students’ attitudes toward SPOCs. The association between these variables as applied to online courses has been demonstrated in prior studies (Harnadi et al., 2024; Ananto et al., 2025). Therefore, we propose the following research hypotheses:

*H1:* Perceived ease of use positively influences perceived usefulness.

*H2:* Perceived ease of use positively influences attitude.

*H3:* Perceived usefulness positively influences attitude.

*H4:* Perceived usefulness negatively influences negative behavior.

*H5:* Attitude positively influences negative behavior.

### 3.2 Task-technology fit (TTF), individual-technology fit (ITF) and online-to-offline fit (OOF)

In this study, we extend TAM with a series of prior factors (i.e., TTF, ITF, and OOF) to consider the interrelationship between the SPOC platform, SPOC tasks, and students.

TTF theory posits that technology effectiveness depends on congruence between task requirements and system capabilities (Furneaux, 2012). The theory has been widely used by information system researchers and has yielded a series of valuable outcomes (Alyoussef, 2023). The mismatch in SPOC,—e.g., overly complex navigation for simple information-seeking tasks or inadequate interactive features for collaborative assignments—create friction, reducing perceived usefulness and ease of use (Zhang L. et al., 2019). In this study, we applied TTF to evaluate the degree of fit between the SPOC platform and students’ assignments in class.

We propose the following hypotheses in connection with TTF:

*H6a*: Task-technology fit positively influences perceived usefulness.

*H6b*: Task-technology fit positively influences perceived ease of use.

ITF is the extent to which the technology (decision guidance) fits the individual (task expertise) (Parkes, 2013; Dhiman and Jamwal, 2023). Students’ effective use of SPOCs depends on factors related to individual-technology fit, including whether learning styles and course characteristics match students’ learning habits and targets. Better learning outcomes and a greater perception of the SPOC platform’s usefulness will arise from students’ increased ability to adjust to the platform’s teaching strategies. Many researchers have applied the theory in their studies, including Dhiman and Jamwal (2023) and Liu K. et al. (2023).

We propose the following further hypotheses related to ITF:

*H7a:* Individual-technology fit positively influences perceived usefulness.

*H7b*: Individual-technology fit positively influences perceived ease of use.

Taking into account the unique characteristics of SPOCs, where students first learn independently online and then engage in conversation with professors in flipped classes. OOF was first introduced by Zhang L. et al. (2019) to evaluate the coherence between online content (e.g., pre-class videos, quizzes) and offline activities (e.g., in-class discussions, hands-on exercises). Students are more likely to have a positive attitude toward the SPOC learning method when they believe there is a better fit between online and offline materials (e.g., online unit tests, assignments, and exams). In this study, we adopt OOF to capture the influence of online and offline conditions.

We propose the following hypotheses regarding OOF:

*H8a*: Online-to-offline fit positively influences perceived usefulness.

*H8b*: Online-to-offline fit positively influences perceived ease of use.

### 3.3 Teachers (T), courses (Cr) and community (Cm)

We also introduce a series of SPOC features to increase model accuracy. In SPOCs, teachers act as primary facilitators by managing both online and flipped offline classes, responsible for designing online-offline integration, facilitating discussions, and providing personalized feedback. Social cognitive theory posits that teacher credibility (expertise, enthusiasm) enhances student self-efficacy, which in turn boosts perceived usefulness of learning activities (Yan et al., 2021). Hence, we apply the variable T to evaluate the quality of teachers in SPOCs.

We propose the following hypotheses related to the role of teachers:

*H9a*: The teacher quality factor positively influences perceived usefulness.

*H9b*: The teacher quality factor positively influences perceived ease of use.

Meanwhile, the courses themselves can also have a dramatic effect on students’ behavior. It may be more likely for students to leave when they enroll in poor-quality SPOCs. A course’s quality is determined by a number of factors, such as its length, difficulty, and content. Deng et al. (2020) and Ucha (2023) both considered course characteristics in their earlier studies.

We propose the following hypotheses regarding course quality:

*H10a*: Course quality positively influences perceived usefulness.

*H10b*: Course quality positively influences perceived ease of use.

Finally, the learning environment can also have a significant impact on students’ learning behavior. The likelihood that a student will learn in a SPOC increases with the number of SPOC courses taken by other students in the immediate vicinity. Meanwhile, SPOCs, like MOOCs, feature an online social academic community where students can collaborate and have discussions. Students who are more engaged in such a community have a strong sense of belonging and are less likely to drop out (Yang et al., 2022; Wang et al., 2023).

We therefore propose the following research hypotheses:

*H11a*: Community quality positively influences perceived usefulness.

*H11b*: Community quality positively influences perceived ease of use.

## 4 Methodology

### 4.1 Survey design

To test the theoretical predictions, a questionnaire survey was conducted. The questionnaire consists of three sections, the first of which explains the study’s purpose and significance. A demographic survey comprising gender, level of education, and college major is covered in the second section; 11 latent variables are measured in the third section. A five-point Likert scale (1: strongly disagree, 5: strongly agree) was used to rate the items (Hasan and Bao, 2022). We adopted mature items from earlier research to ensure representativeness and readability, changing the phrasing to better describe SPOCs’ characteristics. Sources and measurement items are shown in Table 2.

**TABLE 2:** Measurement items and sources.

Construct	Items	Sources
Perceived usefulness (U)	I do not believe SPOCs can improve my learning performance.	(Wang et al., 2020; Wu and Chen, 2017)
	Using SPOCs cannot enhance my learning effectiveness.	
	The knowledge learned in the SPOC is hard to use in real life.	
Perceived ease of use (EU)	Learning to use SPOCs is difficult.	
	It is difficult to become proficient in using SPOCs.	
	The SPOC’s learning method is complex and hard to use.	
	The interaction with SPOCs is unclear and cryptic.	
Task-technology fit (TTF)	SPOCs cannot meet any aspects of my learning requirements.	(Alturki and Aldraiweesh, 2023; Wan et al., 2020)
	The functions of SPOC platform cannot meet my requirements.	
	The quality of SPOCs cannot meet my requirements.	
	I think that using SPOC is unsuited for the way I learn.	
	SPOCs are unable to help me complete online courses.	
Individual-technology fit (ITF)	I cannot complete online courses in SPOCs independently and consciously.	(Wu and Chen, 2017)
	I cannot participate actively in various types of discussion and evaluation in SPOCs.	
	I am lacking in outstanding performance in SPOCs.	
Online-to-offline fit (OOF)	Contents on the SPOC platform cannot fit the requirements of my learning in offline courses.	(Zhang L. et al., 2019)
Course (Cr)	Contents on the SPOC platform cannot fit with my knowledge expansion from offline courses. Contents on the SPOC platform are not suitable for helping me absorb knowledge in offline courses. I find that the duration of SPOCs is unreasonable.	(Deng et al., 2020; Huang et al., 2017)
	I find that the contents of courses are unable to keep pace with the times.	
	I find that completing SPOCs is: 1 = not difficult at all, 5 = extremely difficult.	
Teacher (T)	The teacher doesn’t know the content that he/she teaches very well.	(Huang et al., 2017)
	The teacher cannot make good decisions regarding the depth, scope, and extension of concepts taught.	
	The teacher does a bad job of planning the sequence of concepts taught in class.	
	The interactivity of teacher and students in SPOCs cannot help me understand the content better.	
Community (Cm)	Many of the classmates I pay close attention to do not use SPOCs.	(Yang et al., 2022)
	Other users did not respond actively to my posts.	
	I have a very weak sense of belonging to the SPOC community.	
	I am an unimportant member of the academic community.	
Attitude (A)	I have a negative attitude toward the SPOC platform.	(Alturki and Aldraiweesh, 2023; Hsu et al., 2018)
	I don’t think it is a wise choice to carry out learning through the SPOC platform.	
	I don’t think the use of the SPOC platform meets my various learning needs.	
	I don’t think studying is more interesting with SPOCs.	
	I am unsatisfied with using SPOCs.	
Negative behavior (NB)	It’s unlikely to get a certificate for the SPOC course.	(Hone and El Said, 2016; Wu and Chen, 2017)
	I can’t really finish all of the video courses and tests in the SPOC.	
	I can’t really use all of the SPOC teaching resources.	
	I will decrease my use of SPOCs in the future.	
	I will discontinue my use of SPOCs in the future.	

### 4.2 Data collection

This study’s target population is college students with experience in SPOC courses. In this study we used Questionnaire Star,^1^ a reputable online questionnaire platform, to collect research data, and rewarded the respondents to ensure data quality. The questionnaire URL was disseminated to various university student groups by the experimental group members through personal connections, ensuring that the research subjects covered universities and majors in different regions. After screening out low-quality results, we identified 351 valid samples. Detailed questionnaire content can be found in Supplementry Appendix.

Table 3 shows the demographic distribution of the respondents. Male respondents made up 47.4% of the sample, while female respondents made up 52.6%. Moreover, 52.5% of respondents had undergraduate degrees, 30.6% junior college degrees, and the remainder graduate degrees. Students taking part in this survey were primarily Education (24.3%), Economics/Management (18.3%), or Science (19.1%) majors.

**TABLE 3:** Respondent demographics.

Measure	Items	Frequency	Percentage
Gender	Male	166	47.3
	Female	185	52.7
Education	Normal courses	183	52.1
	Short-cycle courses	108	30.8
	Postgraduates	60	17.1
Field	Engineering	32	9.1
	Education	85	24.2
	Economics/ management	64	18.2
	Science	67	19.1
	Agriculture	21	6.0
	Literature/history/ art	69	19.7
	Medicine	4	1.1
	Philosophy/law	9	2.6

## 5 Analysis

### 5.1 Measurement validation

We evaluated the dependability of the survey data using composite reliability (CR) and Cronbach’s alpha (CA), with results presented in Table 4. Factor loading is utilized to assess whether each item aligns well with the latent variables. All items’ factor loading exceeded the 0.6 standard (Hair et al., 2017), indicating a strong fit. Additionally, CA values all exceed 0.7, indicating adequate dependability and internal consistency (Tavakol and Dennick, 2011).

**TABLE 4:** Reliability and convergent validity testing.

Construct	Measurement item	Factor loading	Cronbach’s alpha	AVE	CR
Perceived usefulness (U)	U1	0.745	0.858	0.549	0.785
	U2	0.703			
	U3	0.773			
Perceived ease of use (EU)	EU1	0.610	0.883	0.575	0.842
	EU2	0.786			
	EU3	0.816			
	EU4	0.803			
Task-technology fit (TTF)	TTF1	0.700	0.926	0.721	0.928
	TTF2	0.862			
	TTF3	0.902			
	TTF4	0.835			
	TTF5	0.928			
Individual-technology fit (ITF)	ITF1	0.808	0.859	0.670	0.859
	ITF2	0.807			
	ITF3	0.840			
Online-to-offline fit (OOF)	OOF1	0.747	0.855	0.672	0.859
	OOF2	0.856			
	OOF3	0.851			
Course (Cr)	Cr1	0.876	0.932	0.823	0.933
	Cr2	0.920			
	Cr3	0.925			
Teacher (T)	T1	0.829	0.897	0.687	0.898
	T2	0.863			
	T3	0.842			
	T4	0.780			
Community (Cm)	Cm1	0.693	0.864	0.622	0.867
	Cm2	0.839			
	Cm3	0.728			
	Cm4	0.879			
Attitude (A)	A1	0.700	0.886	0.568	0.867
	A2	0.829			
	A3	0.700			
	A4	0.727			
	A5	0.802			
Negative behavior (NB)	NB1	0.907	0.943	0.745	0.936
	NB2	0.903			
	NB3	0.823			
	NB4	0.801			
	NB5	0.876			

Discriminant and convergent validity are also considered. As shown in Table 4, the average variance extracted (AVE) ranges from 0.549 to 0.823, exceeding the threshold of 0.5 (Zhang and Zheng, 2021) and illustrating the model’s excellent convergent validity. The Heterotrait-Monotrait ratio (HTMT) and the Fornell-Larcker criterion were used to evaluate the discriminant validity (Tables 5, 6). All HTMT values were less than 0.85 (Hair et al., 2021), indicating strong discriminant validity. Table 6 further supports this by demonstrating that the square root of the AVE (on the diagonal line) exceeds its correlations with other latent variables.

**TABLE 5:** Heterotrait-Monotrait (HTMT) ratio test for discriminant validity.

	U	EU	TTF	ITF	OOF	Cr	T	Cm	A	NB
U	–									
EU	0.529	–								
TTF	0.476	0.536	–							
ITF	0.518	0.534	0.567	–						
OOF	0.56	0.539	0.524	0.458	–					
Cr	0.591	0.632	0.532	0.551	0.637	–				
T	0.46	0.446	0.546	0.343	0.486	0.515	–			
Cm	0.562	0.574	0.512	0.49	0.551	0.761	0.477	–		
A	0.541	0.51	0.533	0.587	0.516	0.602	0.423	0.559	–	
NB	0.527	0.539	0.596	0.558	0.503	0.572	0.416	0.506	0.544	–

**TABLE 6:** Fornell-Larcker criterion test for discriminant validity.

	U	EU	TTF	ITF	OOF	Cr	T	Cm	A	NB
U	0.818									
EU	0.46	0.813								
TTF	0.427	0.484	0.85							
ITF	0.446	0.465	0.508	0.818						
OOF	0.485	0.467	0.469	0.395	0.82					
Cr	0.512	0.552	0.478	0.476	0.551	0.826				
T	0.404	0.397	0.499	0.301	0.427	0.455	0.829			
Cm	0.465	0.48	0.442	0.405	0.457	0.631	0.402	0.755		
A	0.469	0.451	0.486	0.512	0.45	0.527	0.377	0.469	0.783	
NB	0.474	0.494	0.56	0.502	0.453	0.519	0.382	0.439	0.495	0.878

### 5.2 Structural results and hypothesis testing

**FIGURE 2:**
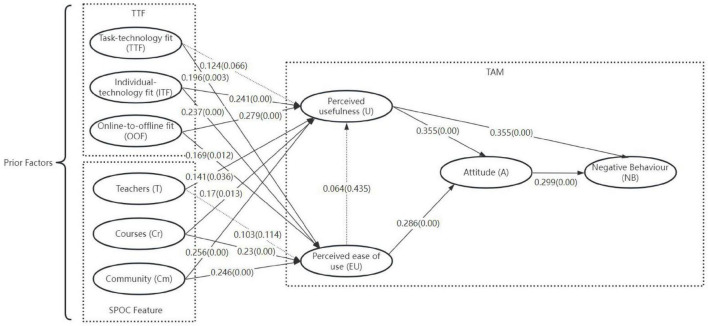
Estimation of research model.

After the accuracy and precision of the measurement model have been verified, the negative learning behavior of SPOC students can be assessed. To assess the validity of the model, we utilized R^2^ values, the effect size (f^2^) and, the Stone-Geisser (Q^2^) predictive relevance, path coefficients and their statistical significance (Hair et al., 2020). R^2^ indicates the model’s interpretive power, while f^2^ demonstrates how dependent latent variables are affected by exogenous latent variables. The predictive relevance of the model is shown by Q^2^. Tables 7, 8 and Figure 2 present the outcome of the structural model.

**TABLE 7:** Predictive relevance analysis.

Construct	R-square	Q-square
Perceived usefulness	0.301	0.348
Perceived ease of use	0.247	0.376
Attitude	0.27	0.345
Negative behavior	0.31	0.309

**TABLE 8:** Empirical results.

Hypotheses	Path	Standard estimate	S. E.	C. R.	*P*	F-square	Supported
H1	EU→U	0.064	0.07	0.781	0.435	0.011	No
H2	U→A	0.355	0.075	4.463	***	0.124	Yes
H3	EU→A	0.286	0.06	3.864	***	0.1	Yes
H4	U→NB	0.355	0.117	4.6	***	0.112	Yes
H5	A→NB	0.299	0.117	4.075	***	0.141	Yes
H6a	TTF→U	0.124	0.073	1.841	0.066	0.002	No
H6b	TTF→EU	0.196	0.082	3	0.003	0.02	Yes
H7a	ITF→U	0.241	0.054	3.299	***	0.027	Yes
H7b	ITF→EU	0.237	0.058	3.485	***	0.03	Yes
H8a	OOF→U	0.279	0.07	3.84	***	0.031	Yes
H8b	OOF→EU	0.169	0.075	2.513	0.012	0.021	Yes
H9a	T→U	0.141	0.053	2.098	0.036	0.023	Yes
H9b	T→EU	0.103	0.06	1.579	0.114	0.006	No
H10a	Cr→U	0.17	0.046	2.472	0.013	0.02	Yes
H10b	Cr→EU	0.23	0.05	3.548	***	0.034	Yes
H11a	Cm→U	0.256	0.072	3.469	***	0.028	Yes
H11b	Cm→EU	0.246	0.078	3.597	***	0.037	Yes

***Represents a statistically significant result with *p* < 0.001.

All R^2^ values for U, EU, A, and NB exceed 0.2, indicating good explanatory power. Additionally, the magnitude of the exogenous latent variables’ f^2^ effect on the four dependent latent variables is displayed in Table 8. Specifically, the f^2^ effect on the U (for EU and TTF) and EU (for T) is low, indicating that these exogenous latent variables have almost no effect on the dependent latent variables. Furthermore, the model’s predictive relevance is shown to be acceptable by the Q^2^ values of U, EU, A, and NB, which are, respectively, 0.348, 0.376, 0.345, and 0.309 and all exceed 0.

Consequently, significantly positive results were found for 14 of the original 17 hypotheses. Specifically, U has the largest impact on A and NB (path coefficient of 0.355), followed by the effect of A on NB (0.299) and EU on A (0.286), proving the adaptability of TAM in this study. OOF has the greatest effect on U (path coefficient of 0.279), followed by Cm, ITF, Cr and T. Cm has the most significant effect on EU (0.246), followed by ITF, Cr, TTF and OOF. Community quality outperforms teacher or course quality in driving both U and EU, emphasizing the need for intentional social design (e.g., facilitated discussions, peer feedback mechanisms). Individual-technology fit matters more than task-technology fit, indicating platforms should prioritize adaptability (e.g., adjustable pacing, multiple content formats) over rigid functionality. Notably, teacher quality had minimal direct effects on U and EU, Platform design and course structure are more immediate levers for enhancing user perceptions.

### 5.3 Re-analysis using fsQCA

This article uses fuzzy-set qualitative comparative analysis to examine the effects of various component combinations on behavioral outcomes, following route analysis of the data. In this study, five independent variables (i.e. TTF, OOF, ITF, T, Cr and Cm) were chosen as the antecedent conditions and NB as the outcome variable. Three fundamental steps in the application of fsQCA are data calibration, truth table generation, and causal condition analysis.

For calibration, all latent variables are converted into fuzzy sets. After calculating three calibration thresholds for each influencing factor (i.e., 95, 50 and 5%), the calibration of the data is completed using fsQCA software. Table 9 shows the necessity analysis outcome for 6 preconditions (TTF, ITF, OOF, Cr, T, Cm, and their absences) with NB as the outcome variable. In general, a variable was deemed required for NB when its consistency level exceeded 0.9 (Ragin, 2000). As indicated in Table 9, there is no prerequisite, because each variable’s consistency score is less than 0.9.

**TABLE 9:** Necessity analysis.

Preconditions	Consistency	Coverage
TTF	0.762 (0.379)	0.809 (0.354)
∼TTF	0.392 (0.796)	0.418 (0.746)
ITF	0.775 (0.443)	0.769 (0.387)
∼ITF	0.382 (0.736)	0.438 (0.742)
OOF	0.732 (0.421)	0.774 (0.392)
∼OOF	0.426 (0.757)	0.455 (0.713)
Cr	0.766 (0.430)	0.770 (0.380)
∼Cr	0.384 (0.740)	0.434 (0.735)
T	0.723 (0.486)	0.726 (0.429)
∼T	0.431 (0.690)	0.488 (0.687)
Cm	0.727 (0.412)	0.772 (0.384)
∼Cm	0.420 (0.755)	0.448 (0.709)

“∼” Denotes “negation.”

The last step is to conduct a sufficiency analysis. First, we set the consistency criterion to 0.8 for screening and the case threshold to 3; these represent the number of samples supporting the configuration, based on the sample size. Next, we choose the items in the screening results that have a PRI threshold of at least 0.7 to choose the path to take. Three sets of solutions—complex, parsimonious, and intermediate—result from this.

Compared to the complex solution, the parsimonious and intermediate solutions are more effective in discerning between core and peripheral scenarios (Fiss, 2011). The configuration results are summed up in Table 10, which also clarifies how the elements are integrated and how each configuration affects SPOC students’ negative learning behavior.

**TABLE 10:** Configurations that affect NB.

Variables	1a	1b	2	3a	3b
TTF	•	•	•	•	•
ITF	•	•	•	⋅	
OOF	•	•		•	•
T	⋅		•	•	•
Cr	⋅	⋅	⋅		⋅
Cm		⋅	•	•	•
Consistency	0.946	0.943	0.929	0.935	0.94
Raw coverage	0.46	0.457	0.451	0.449	0.459
Unique coverage	0.033	0.030	0.025	0.022	0.032
Solution consistency	0.913				
Solution coverage	0.569				

^•^Core causal condition (present). ⋅Peripheral causal condition (present). ^⊗^Core causal condition (absent). Blank space indicates “do not care.”

This study divides the five configurations into three categories of approaches that are successful in affecting students’ negative behavior based on the fsQCA results. First, configurations 1a and 1b are described as a match-dominant driven path with TTF, ITF and OOF as the core conditions, and a sum of coverage of 0.063. Configuration 1a is TTF*ITF*OOF*T*Cr. In this path, students’ negative learning behavior can be affected by the combination of high TTF, ITF, OOF, T and Cr. Configuration 1b is TTF*ITF*OOF*Cr*Cm, or a combination of high TTF, ITF, OOF, Cr and Cm. In configuration 2 (TTF*ITF*T*Cr*Cm), TTF, ITF, T and Cm are the core conditions. In this path, the guidance of teachers and the learning atmosphere may have a significant impact on whether negative learning behavior occurs. Finally, configurations 3a (TTF*ITF*OOF*T*Cm) and 3b (TTF*OOF*T*Cr*Cm) emphasize the impact of students’ learning experience and adaptability to SPOC courses, with TTF, OOF, T and Cm as the core conditions.

## 6 Discussion

This research aims to investigate the significant variables and configurations that affect the negative behavior of college students in SPOCs. By using a mixed-method approach that combines fsQCA and PLS-SEM, this study expands the field of SPOC research. In the post-pandemic era, as online learning gains attention and popularity, leveraging SPOCs to enhance university classes effectiveness becomes increasingly important. While it is technically possible to use PLS-SEM to answer research questions and test hypotheses regarding negative learning behavior (NB) in SPOCs, fsQCA reveals that alternative solutions that employ an ideal combination of prior factors are equally effective in predicting SPOC NB.

Prior to this study, there was a major gap in research on SPOCs’ efficacy. Despite numerous similarities between MOOCs and SPOCs, the latter require special investigation and study because of their “online-to-offline” characteristic. This paper builds upon and supplements the research achievements of L. Zhang L. et al. (2019), and the research data reaffirm their conclusions: students are more likely to drop out of SPOC when there is a low online-offline match. Moreover, this paper also accounts for the intrinsic properties of a series of SPOC courses within the PLS-SEM. The data show that teachers, online courses, and learning communities all have a significant impact on student withdrawal behavior, consistent with the findings of Wang et al. (2022). Among these, the teacher’s ability and attitude, as well as students’ adaptability to SPOC technology, are the two most important factors. Therefore, educators should focus on teacher and student training so that SPOCs may be completely and efficiently employed. This will help to decrease the incidence of SPOC negative behavior among college students and improve learning efficacy in flipped classrooms. Perceived usefulness and perceived ease of use significantly influence students’ attitudes toward SPOCs, which aligns with the Technology Acceptance Model (TAM).

.Notably, perceived ease of use does not exhibit a positive correlation with perceived usefulness—indicating that students do not necessarily view SPOCs as more useful simply because the platform is easy to use. Furthermore, Task-technology fit does not significantly affect perceived usefulness in the context of SPOCs, a finding that contrasts with prior PLS-SEM studies on MOOCs (Wu and Chen, 2017). This divergence may be because MOOCs are typically voluntary learning experiences, where learners actively choose platforms based on personal preferences and perceived utility. In contrast, SPOC participation is often institutionally mandated, making course completion a compulsory requirement, students have limited autonomy in selecting either the learning platform or the subject matter. Since SPOC learners generally engage with only one designated system throughout their coursework, they lack alternative platforms for comparison, thereby dampening the impact of both ease of use and task-technology alignment on perceived usefulness. Additionally, instructors’ teaching competence does not significantly enhance students’ perception of SPOC platform ease of use. This may be because teachers typically do not have the authority to select or customize the SPOC platforms, their influence remains confined to content delivery and pedagogical design rather than technical usability. Consequently, teachers effectiveness becomes decoupled from the perceived ease of use of the platform in SPOC settings. In summary, the dynamics of technology acceptance in SPOCs diverge from those observed in MOOCs due to differences in user autonomy and environmental constraints. These findings suggest that TAM and TTF, while still broadly applicable, requires contextual adaptation when applied to compulsory, institutionally embedded e-learning systems like SPOCs. These findings essentially respond to the first question: what is the relationship between SPOC characteristics and students’ negative learning behavior?

Aside from that, the fsQCA findings suggest that the independent factors are all necessary to avoid negative learning behavior, but no one factor was sufficient. This result supports the claim that the students’ negative behavior is an outcome of multiple factors (Viloria and Lezama, 2019). Notably, the ITF and TTF of SPOC platforms significantly influence various conditional combinations, which is consistent with previous research (Wu and Chen, 2017). This is mainly because individual technology fit and task-technology fit are the most direct perceptions students have when exposed to SPOCs, affecting their initial impressions and attitudes toward SPOCs, thereby influencing their learning behaviors. Therefore, schools should select SPOC platforms that are more aligned with students’ own technical characteristics and learning task requirements. Negative learning behaviors are more likely to arise if the SPOC content differs from the offline classroom and if students have minimal prior experience with SPOC learning (1a, 1b). Meanwhile, a supportive learning environment and advice from teachers can also influence students’ SPOC learning behavior when there are shortcomings or gaps in the SPOC curriculum and students’ former experience (2, 3a, 3b). By comparing the consistency and coverage of different configurations, we can conclude that the primary elements influencing students’ SPOC negative behavior include their learning experience, how easily SPOC course content can be adapted to offline classrooms, and how user-friendly SPOC platforms are. This answers the second research question: which factors are necessarily or sufficiently crucial for SPOC negative learning behavior to occur?

### 6.1 Theoretical implications

This paper makes multiple theoretical contributions. Firstly, Shifting the Paradigm: From Intentions to Negative Behavior. Prior studies mainly focused on students’ intentions and satisfaction with online learning (Hewei and Youngsook, 2022; Zhang et al., 2024), While valuable, this “intentions-centric” paradigm overlooks a critical reality: even when students report high initial intent, many exhibit negative learning behaviors (e.g., procrastination, passive participation, dropout) that undermine actual performance. Our study attempts to consider the problem from the opposite perspective and establish a more targeted model.

Secondly, SPOCs’ hybridity—combining technology, individual differences, and social interaction—demands a more nuanced view of how factors interact to shape NLB (Negative Learning Behavior). We integrated perceived value, SPOC course characteristics (teacher, curriculum, community), and technology matching models, extending early research and improves the realism of the research findings (Wang T. et al., 2021; Zhang L. et al., 2019).

Thirdly, this study apply fsQCAto SPOC learning research, addressing the limitation of variance-based methods like PLS-SEM: their inability to model equifinality (multiple pathways to the same outcome) and causal complexity (interactions between factors). Based on the findings of PLS-SEM, we applied fsQCA to identify the core factors in this respect. While PLS-SEM identified individual predictors (e.g., OOF→U→NB), fsQCA revealed that NB arises not from isolated deficits but from combinations of misfits and contextual failures. For example: Configuration 1: Low TTF + poor course quality + weak community→NB (overwhelm due to technological, pedagogical, and social deficits). These findings deepen theoretical understanding by showing that technology adoption theories must move beyond linear effects to embrace configurational thinking.

### 6.2 Managerial contribution

The results have significant consequences for educators—specifically in helping them conduct SPOC courses better. The most significant individual element is the effective synergy between offline flipped classroom and SPOC course content. For example, for course designers, prioritize online-offline fit (OOF) by aligning pre-class online modules (e.g., videos, quizzes) with in-class flipped activities (e.g., debates) to avoid redundancy and reinforce learning coherence.

Meanwhile, the fsQCA results revealed that the presence or absence of task-technology fit (TTF, ITF, OOF) and certain SPOC features (T, Cm) can equally lead to students’ negative learning behavior, depending on how they combine with other factors. For university administrators, it is important to monitor configuration risks (such as low ITF+impoverished communities) for early intervention.

### 6.3 Limitations and future directions

This study possesses some limitations that, in turn, suggest future research directions. Firstly, the questionnaire is distributed mostly within the researchers’ social circle. Although data was collected from all over the country, the regional distribution is uneven, with the majority of participants coming from the Yangtze River Delta region. Future studies should adopt stratified sampling or random sampling methods, incorporating a wider range of geographical regions and a larger sample size as these factors may influence the model’s applicability. Secondly, students’ learning behavior may evolve with course progress, but such dynamic changes have not been taken into account here. Additional longitudinal studies on students’ learning behavior in SPOCs could be conducted in the future. Thirdly, alternative approaches with greater predictive potential, such as artificial intelligence, may provide more in-depth insights.

## 7 Conclusion

This study understanding of negative learning behavior (NLB) in Small Private Online Courses (SPOCs) by investigating structural equation modeling and fsQCA to unpack causal complexity.. Moving beyond prior linear models, we identified direct drivers (perceived usefulness, attitude) and configurational pathways (e.g., low online-offline fit + poor community quality) that jointly predict NLB among Chinese undergraduates. Through this study, we have identified the main pathways influencing students’ SPOC negative behavior and revealed the core configuration of factors. Ultimately, this study calls for a reimagining of online education as a dynamic, context-sensitive system—one where student behavior is understood as a product of interconnected factors, not isolated choices. By centering NLB and embracing complexity, we provide a roadmap for building more resilient, inclusive SPOCs learning environments that empower students to thrive

## Data Availability

The original contributions presented in this study are included in this article/Supplementary material, further inquiries can be directed to the corresponding author.

## Footnotes

1
www.wjx.cn

## References

[B1] Alario-Hoyos C. Estévez-Ayres I. Delgado Kloos C. Villena-Román J. (2017). “From MOOCs to SPOCs and from SPOCs to flipped classroom,” in *European Conference on technology enhanced learning*, (Cham: Springer International Publishing), 347–354.

[B2] Alturki U. Aldraiweesh A. (2023). Integrated TTF and self-determination theories in higher education: The role of actual use of the massive open online courses. *Front. Psychol.* 14:1108325. 10.3389/fpsyg.2023.1108325 36818124 PMC9933983

[B3] Alyoussef I. Y. (2023). Acceptance of e-learning in higher education: The role of task-technology fit with the information systems success model. *Heliyon* 9:e13751. 10.1016/j.heliyon.2023.e13751 36845042 PMC9938001

[B4] An S. Li W. Hu J. Ma L. Xu J. (2017). “Research on the reform of flipped classroom in computer science of university based on SPOC,” in *Proceedings of the 2017 12th International Conference on Computer Science and Education (ICCSE)*, (Houston, TX: IEEE), 621–625.

[B5] Ananto T. S. Muhamad E. Muhamad F. (2025). Dataset on technology acceptance in E-learning: A PLS-SEM analysis using extended TAM among undergraduate students in Indonesia. *Telem. Inform. Rep.* 18:100192. 10.1016/j.teler.2025.100192

[B6] Astrachan C. B. Patel V. K. Wanzenried G. (2014). A comparative study of CB-SEM and PLS-SEM for theory development in family firm research. *J. Fam. Bus. Strategy* 5 116–128. 10.1016/j.jfbs.2013.12.002

[B7] Baturay M. H. (2015). An overview of the world of MOOCs. *Procedia-Soc. Behav. Sci.* 174 427–433. 10.1016/j.sbspro.2015.01.685

[B8] Deng R. Benckendorff P. Gannaway D. (2020). Linking learner factors, teaching context, and engagement patterns with MOOC learning outcomes. *J. Comp. Assis. Learn.* 36 688–708. 10.1111/jcal.12437

[B9] Dhiman N. Jamwal M. (2023). Tourists’ post-adoption continuance intentions of chatbots: Integrating task–technology fit model and expectation–confirmation theory. *Foresight* 25 209–224. 10.1108/FS-10-2021-0207

[B10] Du M. (2021). “Self-regulated learning model in SPOC of blended learning based on online education platform,” in *Proceedings of the 2021 2nd International Conference on Information Science and Education (ICISE-IE)*, (China), 10.1109/ICISE-IE53922.2021.00279

[B11] Esther C. H. Peter G. M. Floris M. V. B. Marlies E. J. R. (2022). Design and first impressions of a small private online course in clinical workplace learning: Questionnaire and interview study. *JMIR Med. Educ.* 8:e29624. 10.2196/29624 35389362 PMC9030912

[B12] Filius R. M. de Kleijn R. A. Uijl S. G. Prins F. J. van Rijen H. V. Grobbee D. E. (2018). Strengthening dialogic peer feedback aiming for deep learning in SPOCs. *Comp. Educ.* 125 86–100. 10.1016/j.compedu.2018.06.004

[B13] Fiss P. C. (2011). Building better causal theories: A fuzzy set approach to typologies in organization research. *Acad. Manag. J.* 54 393–420. 10.5465/amj.2011.60263120

[B14] Fox A. (2013). From MOOCs to SPOCs. *Commun. ACM* 56 38–40. 10.1145/2535918

[B15] Furneaux B. (2012). “Task-technology fit theory: A survey and synopsis of the literature,” in *Information systems theory. integrated series in information systems*, Vol. 28 eds Dwivedi Y. Wade M. Schneberger S. (New York, NY: Springer).

[B16] Gligor D. Bozkurt S. (2020). fsQCA versus regression: The context of customer engagement. *J. Retail. Consumer Services* 52:101929. 10.1016/j.jretconser.2019.101929

[B17] Guo P. (2017). MOOC and SPOC, which one is better? *Eur. J. Mathem. Sci. Technol. Educ.* 13 5961–5967. 10.12973/eurasia.2017.01044a

[B18] Hair J. F. Jr. Howard M. C. Nitzl C. (2020). Assessing measurement model quality in PLS-SEM using confirmatory composite analysis. *J. Bus. Res.* 109 101–110. 10.1016/j.jbusres.2019.11.069

[B19] Hair J. Jr. Hult G. T. M. Ringle C. M. Sarstedt M. (2021). *A primer on partial least squares structural equation modeling (PLS-SEM).* Thousand Oaks, CA: Sage Publications.

[B20] Hair J. Hollingsworth C. L. Randolph A. B. Chong A. Y. L. (2017). An updated and expanded assessment of PLS-SEM in information systems research. *Indust. Manag. Data Syst.* 117 442–458. 10.1108/IMDS-04-2016-0130

[B21] Harnadi B. Widiantoro A. D. Prasetya F. X. H. (2024). Investigating the behavioral differences in the acceptance of MOOCs and E-learning technology. *Comp. Hum. Behav. Rep.* 14:100403. 10.1016/j.chbr.2024.100403

[B22] Hasan N. Bao Y. (2022). A mixed-method approach to assess users’ intention to use mobile health (mHealth) using PLS-SEM and fsQCA. *Aslib J. Inform. Manag.* 74 589–630. 10.1108/AJIM-07-2021-0211

[B23] Hewei T. Youngsook L. (2022). Influencing factors of online course learning intention of undergraduates majoring in art and design: Mediating effect of flow experience. *SAGE Open* 12:21582440221134004. 10.1177/21582440221134004

[B24] Hone K. S. El Said G. R. (2016). Exploring the factors affecting MOOC retention: A survey study. *Comp. Educ.* 98 157–168. 10.1016/j.compedu.2016.03.016

[B25] Hsu J.-Y. Chen C.-C. Ting P.-F. (2018). Understanding MOOC continuance: An empirical examination of social support theory. *Interact. Learn. Environ.* 26 1100–1118. 10.1080/10494820.2018.1446990

[B26] Huang L. Zhang J. Liu Y. (2017). Antecedents of student MOOC revisit intention: Moderation effect of course difficulty. *Intern. J. Inform. Manag.* 37 84–91. 10.1016/j.ijinfomgt.2016.12.002

[B27] Jiang J. F. Yin H. D. (2024). Project-based deep learning strategies based on SPOC platform. *Educ. Sci. Res.* 35, 57–64. 10.3969/j.issn.1009-718X.2024.05.009

[B28] Jiang L. Liang X. (2023). Influencing factors of Chinese EFL students’ continuance learning intention in SPOC-based blended learning environment. *Educ. Inform. Technol.* 10.1007/s10639-023-11734-4 [Epub ahead of print]. 37361808 PMC10043519

[B29] Kelana B. Riskinanto A. Hayati I. N. (2017). “SPOC adoption in accounting course among Indonesian undergraduate students: A case study,” in *Paper presented at the 2017 International Conference on Sustainable Information Engineering and Technology (SIET)*, (New York, NY: IEEE).

[B30] King W. R. He J. (2006). A meta-analysis of the technology acceptance model. *Inform. Manag.* 43 740–755. 10.1016/j.im.2006.05.003

[B31] Kumar S. Sahoo S. Lim W. M. Kraus S. Bamel U. (2022). Fuzzy-set qualitative comparative analysis (fsQCA) in business and management research: A contemporary overview. *Technol. Forecast. Soc. Change* 178:121599. 10.1016/j.techfore.2022.121599

[B32] Lin X. Zhan Z. Zhang X. Xiong J. (2023). Exploring the effectiveness of a SPOC learning analytics system based on attribution theory: Evaluation framework and educational experiment. *IEEE Trans. Learn. Technol.* 17 98–111. 10.1109/TLT.2023.3268276

[B33] Liu K. Yao J. Tao D. Yang T. (2023). Influence of individual-technology-task-environment fit on university student online learning performance: The mediating role of behavioral, emotional, and cognitive engagement. *Educ. Inform. Technol.* 28 15949–15968. 10.1007/s10639-023-11833-2 37361766 PMC10157568

[B34] Liu X. Zhang C. Wu J. (2023). Explaining consumers’ continuous purchase intention toward subscriber-based knowledge payment platforms: Findings from PLS-SEM and fsQCA. *Aslib J. Inform. Manag.* 76 189–211. 10.1108/ajim-08-2022-0359

[B35] Marangunić N. Granić A. (2015). Technology acceptance model: A literature review from 1986 to 2013. *Univ. Access Inform. Soc.* 14 81–95. 10.1007/s10209-014-0348-1

[B36] Nejkovic V. Tosic M. (2018). Exploring factors for effective use of online information in SPOC within the engineering education. *Comp. Appl. Eng. Educ.* 26 1457–1469. 10.1002/cae.21991

[B37] Parkes A. (2013). The effect of task–individual–technology fit on user attitude and performance: An experimental investigation. *Decision Supp. Syst.* 54 997–1009. 10.1016/j.dss.2012.10.025

[B38] Ragin C. C. (2000). *Fuzzy-set social science*, vol. 30. Chicago: University of Chicago Press, 291–2.

[B39] Reich J. Ruiperez-Valiente J. A. (2019). The MOOC pivot. *Science* 363 130–131. 10.1126/science.aav7958 30630920

[B40] Shi H. Liu M. Zhu Z. Zhang S. (2018). “Research on the learning willingness of engineering college students in SPOC flipped class,” in *Proceedings of the 2018 3rd International Conference on Education, E-learning and Management Technology (EEMT 2018)*, (Dordrecht: Atlantis Press), 51–57.

[B41] Spies R. Grobbelaar S. Botha A. (2020). A scoping review of the application of the task-technology fit theory. *Responsible Design Implement. Use Inform. Commun. Technol.* 12066 397–408. 10.1007/978-3-030-44999-5_33

[B42] Tao D. Fu P. Wang Y. Zhang T. Qu X. (2022). Key characteristics in designing massive open online courses (MOOCs) for user acceptance: An application of the extended technology acceptance model. *Interact. Learn Environ.* 30 882–895. 10.1080/10494820.2019.1695214

[B43] Tavakol M. Dennick R. (2011). Making sense of Cronbach’s alpha. *Intern. J. Med. Educ.* 2 53–55. 10.5116/ijme.4dfb.8dfd 28029643 PMC4205511

[B44] Ucha C. R. (2023). Role of course relevance and course content quality in MOOCs acceptance and use. *Comp. Educ. Open* 5:100147. 10.1016/j.caeo.2023.100147

[B45] Van Alten D. C. Phielix C. Janssen J. Kester L. (2019). Effects of flipping the classroom on learning outcomes and satisfaction: A meta-analysis. *Educ. Res. Rev.* 28:100281. 10.1016/j.edurev.2019.05.003

[B46] Viloria A. Lezama O. B. P. (2019). Mixture structural equation models for classifying university student dropout in Latin America. *Proc. Comp. Sci.* 160 629–634. 10.1016/j.procs.2019.11.036

[B47] Wan L. Xie S. Shu A. (2020). Toward an understanding of university students’ continued intention to use MOOCs: When UTAUT model meets TTF model. *SAGE Open* 10:2158244020941858. 10.1177/2158244020941858

[B48] Wang H. Hou X. Liu J. Zhou X. Jiang M. Liao J. (2024a). Framework effect and achievement motivation on college students’ online learning intention-based on technology acceptance model (TAM) and theory of planned behaviour (TPB) model. *Educ. Inform. Technol.* 30 11073–11097. 10.1007/s10639-024

[B49] Wang H. Zhang W. Kong W. Zhang G. Pu H. Wang Y. (2024b). The effects of ‘small private online course+ flipped classroom’teaching on job competency of nuclear medicine training trainees. *BMC Med. Educ.* 24:1542. 10.1186/s12909-024-06579-5 39731074 PMC11681664

[B50] Wang K. Zhu C. Tondeur J. (2021). Using micro-lectures in small private online courses: What do we learn from students’ behavioural intentions? *Technol. Pedagogy Educ.* 30 427–441. 10.1080/1475939x.2020.1832565

[B51] Wang M. Lin H. Du X. Chu Z. Li J. (2022). Analysis of influencing factors of SPOC course teaching effect using structural equation modelling. *Appl. Mathemat. Nonlinear Sci.* 8 2605–2616. 10.2478/amns.2021.2.00314

[B52] Wang T. Lin C.-L. Su Y.-S. (2021). Continuance intention of university students and online learning during the COVID-19 pandemic: A modified expectation confirmation model perspective. *Sustainability* 13:4586. 10.3390/su13084586

[B53] Wang W. Zhao Y. Wu Y. J. Goh M. (2023). Factors of dropout from MOOCs: A bibliometric review. *Library Hi Tech.* 41 432–453. 10.1108/LHT-06-2022-0306

[B54] Wang Y. Dong C. Zhang X. (2020). Improving MOOC learning performance in China: An analysis of factors from the TAM and TPB. *Comp. Appl. Eng. Educ.* 28 1421–1433. 10.1002/cae.22310

[B55] Wu B. Chen X. (2017). Continuance intention to use MOOCs: Integrating the technology acceptance model (TAM) and task technology fit (TTF) model. *Comp. Hum. Behav.* 67 221–232. 10.1016/j.chb.2016.10.028

[B56] Wut T. M. Xu J. Lee S. W. Lee D. (2022). University student readiness and its effect on intention to participate in the flipped classroom setting of hybrid learning. *Educ. Sci.* 12:442. 10.3390/educsci12070442

[B57] Yan M. Raffaele F. Matthew G. (2021). Continuance intention of online technologies: A systematic literature review. *Intern. J. Inform. Manag.* 58:102315. 10.1016/j.ijinfomgt.2021.102315

[B58] Yang Z. Zhou Q. Chiu D. K. Wang Y. (2022). Exploring the factors influencing continuous usage intention of academic social network sites. *Online Inform. Rev.* 46 1225–1241. 10.1108/oir-01-2021-0015

[B59] Zhang J. Sziegat H. Perris K. Zhou C. (2019). More than access: MOOCs and changes in Chinese higher education. *Learn. Med. Technol.* 44 108–123. 10.1080/17439884.2019.1602541

[B60] Zhang L. Shao Z. Pan Z. Feng Y. (2019). “*Examining individuals’ utilization of SPOC: Extending the task-technology fit model with online and offline perspective*”, in Pacific Asia conference on information systems 2019 Proceedings, Atlanta, GA, 202.

[B61] Zhang Y. Zhang X. Meng Z. (2024). Effect of interactive immediacy on online learning satisfaction of international students in Chinese universities: The chain mediating role of learning interest and academic engagement. *Acta Psychol.* 244:104202. 10.1016/j.actpsy.2024.104202 38430727

[B62] Zhang Z. Zheng L. (2021). Consumer community cognition, brand loyalty, and behaviour intentions within online publishing communities: An empirical study of Epubit in China. *Learned Publishing* 34 116–127. 10.1002/leap.1327

