# Supplementary material for: Negative learning behavior in small private online courses (SPOCs): findings from PLS-SEM and fsQCA
Source: Front Psychol. 2025 Dec 17;16:1634957. doi: 10.3389/fpsyg.2025.1634957 (PMC12753872; doi:10.3389/fpsyg.2025.1634957)
Supplement: Supplementary file 1 [file Data_Sheet_1.docx]

**Appendix: Survey Questionnaire**

*Hello! Thank you for participating in this survey, which aims to better understand the factors the influence college students’ negative learning behaviour in SPOC. Please take a few minutes to complete the following questions. Your honest responses will help inform our research and contribute to the development of relevant initiatives. All your information and data will be protected and used* ***only*** *for this study.*

*To indicate your answer please* **CIRCLE** *the number alongside the answer that comes closest to your opinion or write your answer in the space provided.*

**Please indicate your information.**

Name(optional):

Age:

Gender:

Email Address(optional):

Education level: 1. High school diploma; 2. Bachelor’s degree; 3. Master’s degree; 4. Doctoral degree; 5. Associate degree.

Discipline: 1. Engineering; 2. Education; 3. Economics/Management; 4. Science; 5. Agriculture; 6. Literature/History/Art; 7. Medicine; 8. Philosophy/Law

*The following questions are based on the Likert 5-level scale. Please choose your level of agreement based on the viewpoint expressed in the question: 1. Completely disagree; 2. Disagree; 3. Neutrality; 4. Identification; 5. Fully agree.* ***Please try to avoid selecting the "neutral" option as much as possible.***

| **Q1: Perceived usefulness** | | | | | |
| --- | --- | --- | --- | --- | --- |
| I do not believe SPOCs can improve my learning performance. | 1 | 2 | 3 | 4 | 5 |
| Using SPOCs cannot enhance my learning effectiveness. | 1 | 2 | 3 | 4 | 5 |
| The knowledge learned in the SPOC is hard to use in real life. | 1 | 2 | 3 | 4 | 5 |
| **Q2: Perceived ease of use** | | | | | |
| Learning to use SPOCs is difficult. | 1 | 2 | 3 | 4 | 5 |
| It is difficult to become proficient in using SPOCs. | 1 | 2 | 3 | 4 | 5 |
| The SPOC’s learning method is complex and hard to use | 1 | 2 | 3 | 4 | 5 |
| The interaction with SPOCs is unclear and cryptic. | 1 | 2 | 3 | 4 | 5 |
| **Q3: Task-technology fit** | | | | | |
| SPOCs cannot meet any aspects of my learning requirements. | 1 | 2 | 3 | 4 | 5 |
| The functions of SPOC platform cannot meet my requirements. | 1 | 2 | 3 | 4 | 5 |
| The quality of SPOCs cannot meet my requirements. | 1 | 2 | 3 | 4 | 5 |
| I think that using SPOC is unsuited for the way I learn. | 1 | 2 | 3 | 4 | 5 |
| SPOCs are unable to help me complete online courses. | 1 | 2 | 3 | 4 | 5 |
| **Q4: Individual-technology fit** | | | | | |
| I cannot complete online courses in SPOCs independently and consciously. | 1 | 2 | 3 | 4 | 5 |
| I cannot participate actively in various types of discussion and evaluation in SPOCs. | 1 | 2 | 3 | 4 | 5 |
| I am lacking in outstanding performance in SPOCs. | 1 | 2 | 3 | 4 | 5 |
| **Q5: Online-to-offline fit** | | | | | |
| Contents on the SPOC platform cannot fit the requirements of my learning in offline courses. | 1 | 2 | 3 | 4 | 5 |
| Contents on the SPOC platform cannot fit with my knowledge expansion from offline courses. | 1 | 2 | 3 | 4 | 5 |
| Contents on the SPOC platform are not suitable for helping me absorb knowledge in offline courses. | 1 | 2 | 3 | 4 | 5 |
| **Q6: Course** | | | | | |
| I find that the duration of SPOCs is unreasonable. | 1 | 2 | 3 | 4 | 5 |
| I find that the contents of courses are unable to keep pace with the times. | 1 | 2 | 3 | 4 | 5 |
| I find that completing SPOCs is: 1 = not difficult at all, 5 = extremely difficult. | 1 | 2 | 3 | 4 | 5 |
| **Q7: Teacher** | | | | | |
| The teacher doesn’t know the content that he/she teaches very well. | 1 | 2 | 3 | 4 | 5 |
| The teacher cannot make good decisions regarding the depth, scope, and extension of concepts taught. | 1 | 2 | 3 | 4 | 5 |
| The teacher does a bad job of planning the sequence of concepts taught in class. | 1 | 2 | 3 | 4 | 5 |
| The interactivity of teacher and students in SPOCs cannot help me understand the content better. | 1 | 2 | 3 | 4 | 5 |
| **Q8: Community** | | | | | |
| Many of the classmates I pay close attention to do not use SPOCs. | 1 | 2 | 3 | 4 | 5 |
| Other users did not respond actively to my posts. | 1 | 2 | 3 | 4 | 5 |
| I have a very weak sense of belonging to the SPOC community. | 1 | 2 | 3 | 4 | 5 |
| I am an unimportant member of the academic community. | 1 | 2 | 3 | 4 | 5 |
| **Q9: Attitude** | | | | | |
| I have a negative attitude towards the SPOCs platform. | 1 | 2 | 3 | 4 | 5 |
| I don’t think it is a wise choice to carry out learning through the SPOC platform. | 1 | 2 | 3 | 4 | 5 |
| I don’t think the use of the SPOC platform meets my various learning needs. | 1 | 2 | 3 | 4 | 5 |
| I don’t think studying is more interesting with SPOCs. | 1 | 2 | 3 | 4 | 5 |
| I am unsatisfied with using SPOCs. | 1 | 2 | 3 | 4 | 5 |
| **Q10: Nevegate learning behaviour** | | | | | |
| It’s unlikely that I’ll be able to get a certificate for the course. | 1 | 2 | 3 | 4 | 5 |
| I can't really finish all of the video courses and tests in the SPOC. | 1 | 2 | 3 | 4 | 5 |
| I can't really use all of the SPOC teaching resources. | 1 | 2 | 3 | 4 | 5 |
| I intend to stop using SPOCs in the future. | 1 | 2 | 3 | 4 | 5 |
| I will decrease or discontinue my use of SPOCs in the future. | 1 | 2 | 3 | 4 | 5 |

*Thank you for completing the English questionnaire! Your feedback is valuable to us. Have a great day!*

**Note:** This is a paper version of the survey questionnaire for this study. We have published an electronic version of the questionnaire with the same content on the Questionnaire Star platform (www.wjx.cn), but the style may be different.
